# Anti-inflammatory agents as modulators of the inflammation in adipose tissue: A systematic review

**DOI:** 10.1371/journal.pone.0273942

**Published:** 2022-09-01

**Authors:** Sara Sayonara da Cruz Nascimento, Jaluza Luana Carvalho de Queiroz, Amanda Fernandes de Medeiros, Ana Clara de França Nunes, Grasiela Piuvezam, Bruna Leal Lima Maciel, Thaís Souza Passos, Ana Heloneida de Araújo Morais

**Affiliations:** 1 Nutrition Postgraduate Program, Center for Health Sciences, Federal University of Rio Grande do Norte, Natal, RN, Brazil; 2 Biochemistry and Molecular Biology Postgraduate Program, Biosciences Center, Federal University of Rio Grande do Norte, Natal, RN, Brazil; 3 Public Health Postgraduate Program, Center for Health Sciences, Federal University of Rio Grande do Norte, Natal/RN, Brazil; 4 Department of Public Health, Center for Health Sciences, Postgraduate Program in Public Health, Federal University of Rio Grande do Norte, Natal/RN, Brazil; 5 Department of Nutrition, Federal University of Rio Grande do Norte, Natal, RN, Brazil; Vall d’Hebron Institut de Recerca, SPAIN

## Abstract

Obesity is characterized by an adipose tissue mass expansion that presents a risk to health, associated with a chronic increase in circulating inflammatory mediators. Anti-inflammatory agents are an obesity alternative treatment. However, the lack of effective agents indicates the need to assess the mechanisms and identify effective therapeutic targets. The present work identified and described the mechanisms of action of anti-inflammatory agents in adipose tissue in experimental studies. The review was registered in the International Prospective Registry of Systematic Reviews (PROSPERO—CRD42020182897). The articles’ selection was according to eligibility criteria (PICOS). The research was performed in PubMed, ScienceDirect, Scopus, Web of Science, VHL, and EMBASE. The methodological quality evaluation was assessed using SYRCLE. Initially, 1511 articles were selected, and at the end of the assessment, 41 were eligible. Among the anti-inflammatory agent classes, eight drugs, 28 natural, and five synthetic compounds were identified. Many of these anti-inflammatory agents act in metabolic pathways that culminate in the inflammatory cytokines expression reduction, decreasing the macrophages infiltration in white and adipose tissue and promoting the polarization process of type M1 to M2 macrophages. Thus, the article clarifies and systematizes these anti-inflammatory agents’ mechanisms in adipose tissue, presenting targets relevant to future research on these pathways.

## Introduction

Obesity is a growing public health problem that has reached pandemic proportions [1]. According to the World Health Organization (WHO) data, the number of people with this comorbidity has shown significant and constant growth, tripling in the last 20 years [2].

Obesity is characterized by an expansion of adipose tissue and changes in body distribution. This accumulation is associated with a chronic increase in circulating concentrations of inflammatory mediators, including several acute-phase inflammatory proteins, pro, and inflammatory cytokines, adhesion molecules, and pro-thrombotic molecules, resulting in a low-grade, chronic local, and systemic inflammation [3–7].

The normal adipose tissue (AT) also forms about 10–15% of the macrophages. These cells are classified into two types, with an anti-inflammatory phenotype predominant in healthy AT, called M2 or “alternatively activated.” However, in humans and rodents with obesity, there is a considerable macrophage infiltration in response to the local release of monocyte chemoattractant protein-1 (MCP-1). Thus, in the state of obesity, macrophages make up about 50% of the population of interstitial cells, and their phenotype is changed to a pro-inflammatory state, called M1 or “classically activated [8–10]. These macrophages are characterized by the secretion of pro-inflammatory cytokines, resulting in tissue inflammation, and inducing several pathological effects [8].

The classic pro-inflammatory responses of M1 macrophages are dependent on toll-like receptors (TLRs) and on the activation of nuclear factor kappa B/c-Jun N-terminal kinases (NFκB/JNK), involving the phosphorylation of the I kappa B kinase (IκB) inhibitor by the IκB kinase complex (IKK-α, IKK-β, and IKK-γ), and inducing the production of inflammatory cytokines [11, 12]. Conversely, the activation of M2 macrophages leads to the recruitment of peroxisome proliferator-activated gamma receptor (PPAR-γ) or other transcription factors, resulting in an anti-inflammatory state [13–15].

Oxidative stress results from increased reactive oxygen species (ROS) production and causes tissue damage and pathological conditions, such as inflammation [16, 17]. This increase has been associated with increased pro-inflammatory cytokines in white adipose tissue (WAT) and reduced adiponectins [16]. Oxidative stress can cause damage by increasing the expression of Akt, extracellular signal-regulated kinase (ERK), signal transducers and activators of transcription (STAT), and JNK, which have been identified as proteins promoting pathologies, including inflammation [17–19].

This scenario leads to a pro-inflammatory state caused by the production of cytokines as a tumor necrosis factor-alpha (TNF-α), through the activation of the nuclear factor kappa B (NF-kB), as well as interleukin 6 (IL-6) [20–22]. The increase in TNF-α and WAT also favors lipolysis and the release of free fatty acids in the plasma, which triggers lipotoxicity and promotes the development of several diseases mediated by ROS. WAT dysfunction can also alter the expression of transcription factors involved in lipid metabolisms, such as sterol regulatory element-binding proteins (SREBP-1c) and PPAR-γ [23–26].

The treatments used for obesity depend mainly on changes in eating habits and lifestyle, being combined, when necessary, with pharmacotherapy and major surgery aiming at weight loss [27]. Drug use is recommended in patients who do not correspond to the changes above mentioned in the first six months and have some chronic disease induced by obesity [28–30]. In these cases, anti-inflammatory agents are presented as an alternative treatment, being studied both in vitro and in vivo in experimental models and clinical trials [31–33].

Thus, the search for strategies aimed at treating inflammation in adipose tissue requires the analysis of the specific mechanisms of action of each treatment used, seeking to direct them more effectively for their use in humans. Therefore, several animal models that share characteristics of human obesity are used to adapt the development of new treatments. According to Lutz [34], although no model recapitulates all aspects of human obesity and its comorbidities, rodent models allow the view of specific mechanisms, being extremely useful to direct the means of treatment through specific pathways. Thus, considering that this systematic review has as its primary objective to evaluate the mechanisms of action of anti-inflammatory agents in obesity and to indicate effective ways in its treatment, only articles with animal models were chosen to expand them in greater detail precision for future clinical studies.

It is also essential to emphasize that the treatment of obesity does not necessarily have to focus on weight loss, considering that up to a third of people with obesity do not have cardiometabolic abnormalities, being defined as patients with metabolically healthy obesity. These patients also have lower levels of inflammatory markers. Thus, treating inflamed tissue in people with obesity is a strategy for improving their health and quality of life [35].

In this perspective, considering the inflammatory aspect, the primary treatment for obesity would be to slow the growth and expansion of adipocytes [36]. In addition to drugs, several bioactive compounds from plants and synthetics have been commonly used to treat obesity regarding action on adipose tissue. These compounds have anti-inflammatory properties and play a fundamental role in treating obesity through their antioxidant and anti-inflammatory properties [37]. The anti-inflammatory action can be achieved by consuming a diet with antioxidant nutrients that reduce ROS levels and components that induce an inflammatory response [38]. However, studies are still needed to assess the effectiveness and selectivity, understanding the different mechanisms of anti-inflammatory agents regarding their functionality in excess adipose tissue [39].

Given the above and considering the search for new alternatives for treating obesity and its direct correlation with inflammation, it is necessary to understand what mechanisms are involved in these compounds’ actions in adipose tissue to improve experimental and clinical studies. Thus, the present study aimed to gather the scientific literature on the subject through a systematic review to answer the following question: "What are the mechanisms of action of anti-inflammatory agents in adipose tissue?"

## Methodology

This systematic review was prepared following the methodological criteria established by the Items of Preferred Reports for Systematic Reviews and Meta-Analysis (PRISMA) [40] and the guidelines of the Systematic Review Center for Animal Laboratory Experimentation–SYRCLE [41].

The protocol for constructing the systematic review was registered in the International Prospective Registry of Systematic Reviews in progress—PROSPERO, under CRD42020182897. The protocol was described by Nascimento et al. [42].

### Search strategy

Searches for articles were performed through electronic searches in January 2022 in PubMed, ScienceDirect, Scopus, Web of Science, Virtual Health Library (VHL), and EMBASE. The databases’ searches were performed through the CAPES Journal Portal of Brazil, with a login from the Federal University of Rio Grande do Norte. Searches were performed according to the strategy described in Table 1.

**Table 1:** Research strategy equation to search for articles in databases to answer the question: What are the action mechanisms of anti-inflammatory agents in adipose tissue?.

Database	PUBMED	SCOPUS	SCIENCE DIRECT	WEB OF SCIENCE	BVS	EMBASE
**Search Strategy**	Inflammation and “adipose tissue” and “Anti-inflammatory agents” and obesity					

### Inclusion and exclusion criteria

#### Inclusion criteria

Original studies were conducted on male rats and mice with overweight or obesity, presenting a control group and treatment with anti-inflammatory agents, drugs, nutraceuticals, and bioactive compounds.

#### Exclusion criteria

Articles that did not describe the mechanism of action, frequency, dose, and time of the experiment were excluded, in addition to documents that were not scientific articles, case reports, and reviews.

### Data extraction process

The studies were initially selected by reading titles, abstracts, and keywords. With Rayyan QCR [43], a specific online server for systematic reviews and references was managed with Mendeley software [44]. A complete reading of the text was then performed to analyze the defined inclusion and exclusion criteria.

The complete reading of the text was performed for data extraction: bibliographic (author, year of publication, and type of study), experimental design (number and type of control groups), characteristics of the animal model (species, lineage, sex, weight, age), exposure (oral diet, time of the experiment, anti-inflammatory used and its mechanism of action, dosage, time of administration, frequency of administration, type of administration and vehicle), and statistical measures used.

The following outcomes were assessed: reduction of inflammatory markers such as body fat, gene expression, and cytokine levels, such as IL-1, IL-6, IL-8, TNF-alpha, leptin, resistin, and inhibition of the cyclooxygenase enzyme (COX-2), induced nitric oxide synthase (iNOS), increased adiponectin levels, reduced expression of the enzyme 5-lipoxygenase (5-LOX), sensitivity to insulin, and reduced PCR.

### Risk of bias and quality evaluation

After reading the articles included in the review, a qualitative methodological assessment was carried out using the SYRCLE [41] Protocol, assessing the risk of bias in experimental studies. The tool has ten questions, with items punctuated with "yes," indicating a low risk of bias, "no," indicating a high risk of bias, or "not clear," indicating uncertain risk. The reviewers were previously trained and calibrated, ensuring greater uniformity in the evaluation, using Cohen’s kappa agreement coefficient.

## Results and discussion

### Selection and characteristics of the studies

Several search strategies were tested to find the best answer to the starting question. Thus, a single strategy was used for all bases, considering that the others were very comprehensive or restricted when evaluating the results obtained. A similarity was observed when comparing the results obtained in the articles between the bases. This confirms that the strategy covered the requirements and had a better effect when compared to the others.

**Fig 1:**
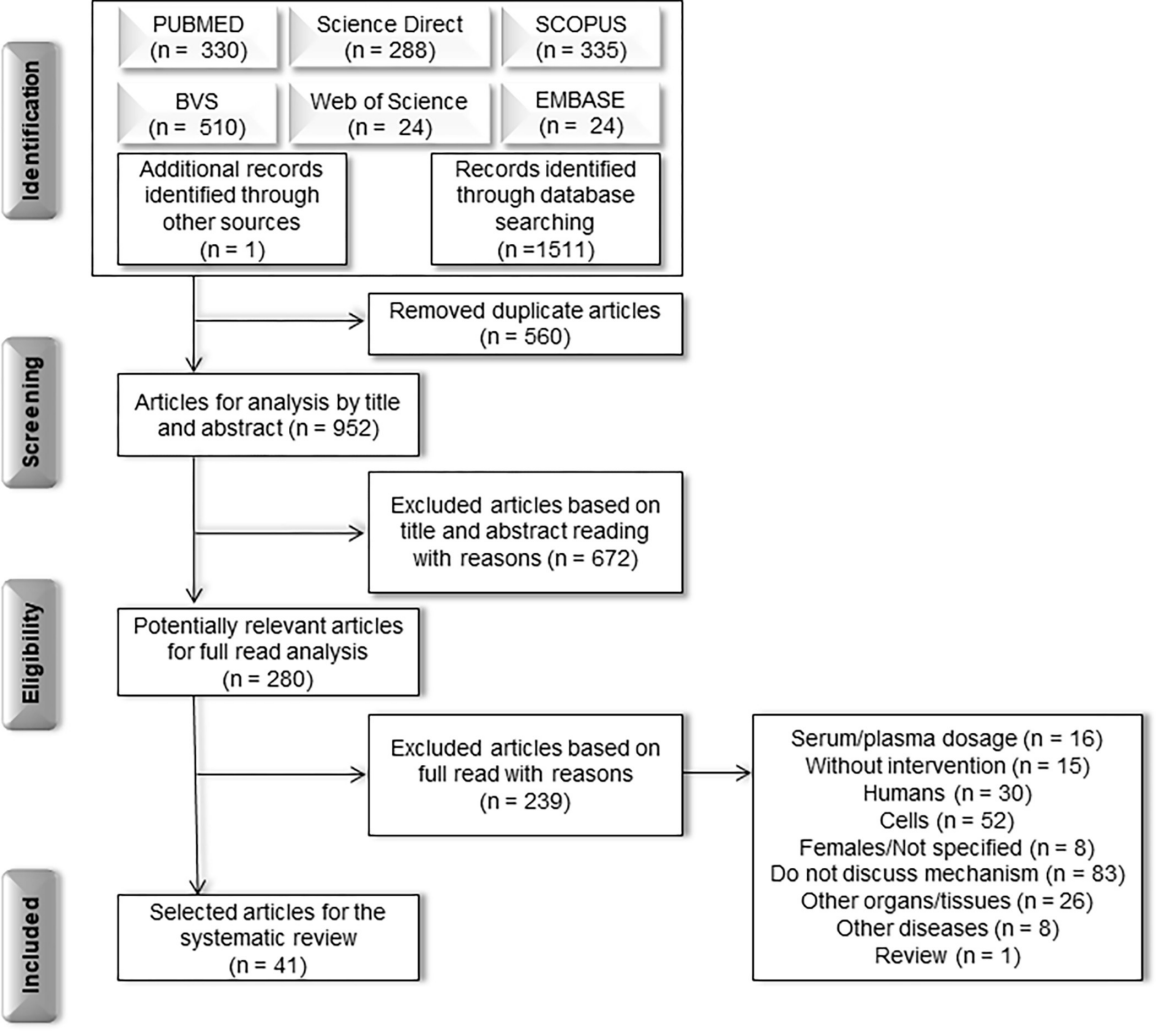
Flowchart for selecting articles based on Preferred Reports for Systematic Reviews and Meta-Analysis (PRISMA) to answer the question: What are the mechanisms of action of anti-inflammatory agents in adipose tissue?.

Initially, 1512 articles were obtained using the search and manual search strategy, 560 of which were duplicated and, therefore, 952 studies for the title and abstract analysis, resulting in 280 articles selected for a complete reading of the text (Fig 1).

Among the excluded articles (Fig 1), 83 did not discuss the mechanism related and had gaps when presenting their results. Many were performed on cells (52), humans (30), other organs and tissues (26), and evaluated serum/plasma dosages (16), not using any intervention (15), treating other diseases (8), or were a review (1). Thus, considering this systematic review’s objectives, 41 experimental studies developed in animals and using anti-inflammatory compounds (drugs, natural compounds, and synthetic compounds) were included.

The data were classified as heterogeneous, considering that, although the compounds could be grouped into specific classes, they differed. In addition, the studies used animals with different lineages and genetic changes. Various dosage forms of the selected markers were observed at different frequencies and follow-up times. Data also presented other measures, formats, and the absence of important information. Thus, it became impracticable to perform a meta-analysis.

### Risk of bias and quality evaluation

**Fig 2:**
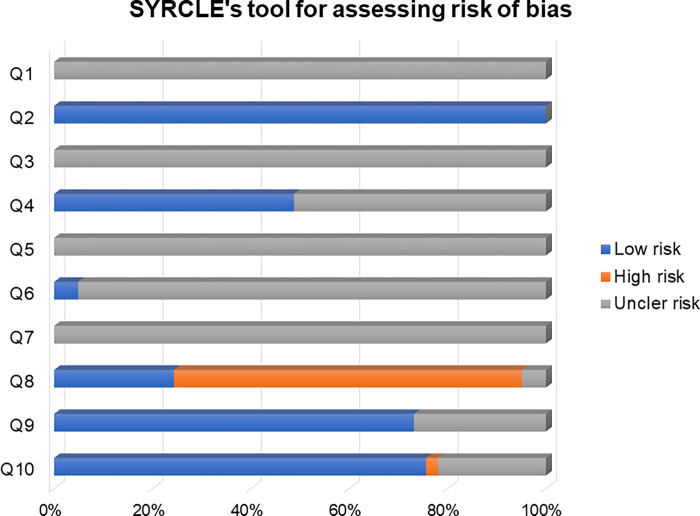
Risk of bias and methodological quality evaluation using the Systematic Review Center for Experimentation in Animal Laboratory (SYRCLE) tool to answer the question: What are the mechanisms of action of anti-inflammatory agents in adipose tissue?.

The trained and calibrated reviewers, ensuring uniformity in the evaluation, presented a Cohen’s kappa concordance coefficient ranging from 0.4 to 1.00. The articles’ methodological quality and risk of bias were assessed using the SYRCLE tool (Fig 2).

No study showed clarity regarding using methods to generate a random allocation sequence (Q1 = uncertain risk of 100% bias) considering the selection bias. All articles presented baseline characteristics and showed animals’ pairing in specific groups (Q2 = 100%). However, none was clear about the evaluators’ blinding to avoid predicting the results (Q3 = uncertain risk of bias 100%). Approximately half of the studies showed the random allocation of animals during the experiment (Q4 = 48.8%), and none declared the researchers’ blindness regarding the treatment for each group (Q5 = 100% uncertain risk of bias).

Regarding the analysis of the results (Q6 and Q7), only two articles reported the random selection of animals, and no article described whether there was the evaluator’s blinding (Q7 = uncertain risk of 100% bias). Most studies did not adequately address data on incomplete results, presenting a high risk of bias (Q8 = 70,7%). Most articles were free from biased reporting of results (Q9 = 73.2%), and no other potential sources of bias were found (Q10 = 75.6%).

The articles selected for the systematic review were ordered by compound: pharmaceuticals, natural and synthetic compounds, in decreasing order according to the SYRCLE score, ranging from 5.5 to 7.5 (Table 2).

**Table 2:** Characteristics of the studies selected for the systematic review separated by categories of anti-inflammatory agents, revealing the outcomes to answer the question: What are the mechanisms of action of anti-inflammatory agents in adipose tissue?.

Author	Compound/Dose and frequency	Time	Animals/Species	Outcomes
**Drugs**				
Hsieh et al. [45]	Colecoxib—30 mg/kg/day	12 weeks	Sprague-Dawley Rats	× COX-2 ↓ Adipocyte hypertrophy ↓CD68 ↓TNF-α ↓PPAR-γ and CEBP-α
Ma et al. [46]	Dextran– 5 mg/kg/day	24 h	C57BL/6J mice	↓CLS ↓MCP-1 ↓TNF-α, IL-6 ↓NF-kB
Ndisang & A. Jadhav [47]	Hemin—30 mg/kg/day	8 weeks	Zucker‐lean (ZL) and ZDF rats	℗ AMPK ↓℗ JNK ↓ Serum levels of inflammatory cytokines ↓MCP-1 ↑ LPL, IRS-1, GLUT-4, PKB/AKT, Adiponectin
Furuya et al. [12]	Atorvastatin—0,1% chow (wt/wt)	4 weeks	CD1 mice	↓TNF-α, IL-6, NF-kB, IKK-α/β ↑GLUT-4
Okada et al. [48]	Pioglitazone (TZD) - 0,001% chow	4 weeks	KKAy mice	↑LXB4, IL-10, IL-13 e Arg-1 ↓Prostaglandin 2
Xu et al. [49]	Empagliflozin (TZD) - 3 or 10 mg/kg/day	16 weeks	C57BL/6J mice	◊ AMPK ↑Adiponectin, UCP-1 ↓F4/80, leptina, CLS × SGLT2
Prabhu et al. [50]	Dextran– 0,1, 0,7, or 5 mg/kg/day	4 weeks	C57BL/6J mice	↓CLS, TNF-α, IL-6, IL-1β, CD68, CD11c, CCR2, CCR5, F4/80, CCL3 and CCL5 ↑ CD206
Zhou et al. [51]	(EX-4)2-Fc, an GLP-1 receptor agonist—1,8 mg/kg^-1^	14 days	C57BL/6 mice	↓ IL-1β, TNF-α, IL-6 e MCP-1 ↓ CD11b, F4/80, iNOS, ↑ CD206 ℗ NF-kB, p38, AMPK
**Natural**				
Lee et al. [52]	Metabolaid® (MetA), verbena and hibiscus—50 or 100 mg/kg/day	8 weeks	C57BL/6 mice	↓PPAR-γ e CEBP-α ↓SREBP1c and FAS ↓TNF-α ↑ UCP1 and UCP2 ℗ AMPK
Fenni et al. [53]	Lycopene and tomato powder supplementation—10 mg/kg of chow/day	12 weeks	C57BL/J6 mice	↓℗ p65 and IKB ↓NF-kB and PPAR ↓SREBP-1c and FAS ↓TNF-α, IL-6, CCL2 and CCL5
Abu Bakar et al. [54]	Celastrol—1 or 3 mg/kg/day	8 weeks	Sprague-Dawley rats	↓IL-6, TNF-α, MCP-1, NF-kB, iNOS, CD11c ↑ Arg1
Alsaggar et al. [55]	Silibinin– 50 mg/kg twice a week	8 weeks	C57BL/6 mice	↓F4/80, CD11c, TNF-α, IL-1β ↑IL-10, adiponectin
Han et al. [56]	Berberine– 1,5 mg/kg/day	12 weeks	C57BL/6 mice	↓ F4/80, IL-6, MCP-1 ↑IL-4, CD206, ARG1, CD163 ↑ M1/M2 reason
Choi, et al. [57]	Indole-3-carbinol (I3C) - 0,1% (wt/wt)	10 weeks	C57BL/6N mice	↑SIRT-1, PGC-α, UCP1 and UCP3 ×℗ERK ↓℗TLR2 and 4 and PPARγ2 ↓TNFα, IFNβ and IL-6
Ma et al. [58]	Spermidine– 20 mg/kg	8 weeks	C57BL/6J mice	↓CLS, F4/80 ↓SREBF1c, FAS, PPARγ, IL-1β, IL-6, iNOS, MCP-1, CCL5, leptin ℗ p38 AMPK, p65 NF-kB
Wei et al. [59]	EPA e DHA– 1, 2 and 4% chow (w/w)	12 weeks	C57BL/6J mice	↓IL-6, TNFα, MCP-1, CD11c, FAS, UCP-1 ↓GPR120, PPARγ
Cho et al. [60]	Carvacrol—0,1% chow (w/w)	10 weeks	C57BL/6N mice	↓TNFα, IFNα ↓TLR2, TLR4, Tirap, TRAF6, IRF5, MyD88 and TRIF
Lee et al. [61]	Black Raspberry Seed Oil—50 ou 100% chow	10 weeks	C57BL/6 and C57BL/KsJ‐db/db mice	↓TLR4, NF-κB ↓TNFα and IL-6 ↓F4/80, CD68 and MCP-1
Kim et al. [62]	Resveratrol—0,4% ~400 mg/kg	10 weeks	C57BL/6J mice	↓TNFα, IFNα, INFβ and IL-6 ↓TLR2, TLR4, MyD88, Tirap, TRIF, TRAF6, IRF5, pIRF3 and NF-kB
Oliveira et al. [63]	α-linolenic (ω3) or oleic (ω9) fatty acids - 10% chow	8 weeks	Swiss Mice	↓IL-1β, IL-6 and TNFα ↓℗ IKK, IκBα and JNK ↑IL-10 ◊ GRP120
Ye et al. [64]	Berberine—50 mg/kg/day	14 days	C57BL/6 mice	× Macrophage polarization M1 ↓MCP-1, IL-6 and TNF-α ↓℗ JNK, IKKβ and AKT ↓ NF-kB ↓F4/80, CD206 ↑CD11c
Gao et al. [65]	Rutin—50 mg/kg twice a week	8 weeks	C57BL/6J mice	↓ Adipocyte hypertrophy and CLS ↓TNF-α ↓MCP-1 ↓F4/80, CD11c and CD68 ◊ CD206 and Arg1
Kang et al. [66]	Capsaicin—0,015% chow	10 weeks	C57BL/6J mice	↓MCP-1, TNFα, IL-6, leptin ↑Adiponectin
Veeramani et al. [67]	Lavatera critica (LC) - 50, 100 or 200 mg/kg/day	5 weeks	C57BL/6J mice	↓TNF-α, IL-1β, IL-6, MCP-1 and CD11c ↓iNOS ↑Ym1, arginase and clec7a
Yang et al. [68]	Bitter melon—1 or 3% chow	6 weeks	OLETF rats	↓TNF-α and IL-6 ↓CCL2 ↓JNK and NF-kB
Ying et al. [69]	Interfeton tau—8 μg/kg/day	8 weeks	C57BL/6J mice	↓JNK, NF-kβ and p65 ↓IL-1β, IL-6, TNF-α and macrophage M1 ↑IL-10 and macrophage M2
Zhou et al. [70]	Catalpol—100 mg/kg/day	4 weeks	C57BL/6J mice	↓ Adipocyte size and CSL ↓IL-1β, IL-6, TNF-α ↓MCP-1, iNOS and CD11c ↓JNK, NF-kB and IKKβ ↑IL-10 ↑MGL1, clec7a and MMR
Subramanian et al. [71]	Terminalia chebula Fruit Extract– 50 mg/kg/day	9 weeks	C57BL/6 mice	↓ TNF-α, IL-6, PPARα, CPT-1α, FAS, adiponectin ↑leptin
Yu et al. [72]	Sarsasapogenina– 40 or 80 mg/kg/day	6 weeks	C57BL/6J mice	↓ TNF-α, IL-1β, IL-6, MCP-1, Nos2, COX2, adiponectin, F4/80 and CD68 ↑Arg1, Ym1, Fizz1 and IL-10
Bashir et al. [73]	Fish oil—4, 8 or 16 mg/kg	4 weeks	C57BL/6 mice	↓IL-1β, IL-2, TNF-α and IFN-γ ↓NF-kB, TLR4 ↓CD86
Bettaieb et al. [74]	Flavan-3-ol (−)-epicatechin—20 mg/kg/day	15 weeks	C57BL/6J mice	↓TNF-α, MCP-1 and F4/80 ↓pIKK, IκB, p65 and NF-κB ↓NOX4, p47
Guo et al. [75]	Cianidin-3-glucoside—0,2% chow	5 weeks	C57BL/6J, C57BL/Ks db/db and db/+ mice	↓TNF-α and IL-6 ↓F4/80, CD11c and MCP-1 ↓JNK and FoxO1
Kwon et al. [76]	Coptidis Rhizoma– 200 or 400 mg/kg/day	10 weeks	C57BL/6 mice	↓CD11c, TNF-α, F4/80, CCL2, CCL4 and CCL5 ↑CD206,
Lee et al. [77]	Broussonetia papyrifera root bark extract—40 mg/kg/day	7 days	C57BL/6 mice	↓NF-kB, IL-1β, iNOS ↑AMPK
Perdicaro et al. [78]	Quercetin– 20 mg/kg/day	6 weeks	Sprague Dawley rats	↑PPARγ/CEBPα, FAS, adiponectin ↓TLR4, CD68, MCP-1, JNK
Ramalho et al. [79]	Eicosapentaenoic Acid– 36 g/kg chow	11 weeks	C57BL/6 mice	↓Alox5, Myd88, Stat1, Ccr5, Card11 and LTB4 ↓miR-146b, miR-125b-5p, miR-30a-5p and miR-143-3p
**Synthetics**				
Dinh et al. [80]	Bardoxolone methyl (BARD) - 10 mg/kg/day	21 weeks	C57BL/6J mice	↓TNF-α and F4/80 ↓ Adipocyte size and CSL ↓STAT3, AKT, ERK, JNK
Wang et al. [81]	Sodium buryate—1 g/kg every two days	6 weeks	C57BL/6J and C57BL/6J db/+ mice	↓TNF-α, IL-1β, IL-6 and INF-γ ↓CD68, F4/80 ↓MCP-1, NLRP3 and caspase 1
Bak et al. [82]	Licochalcones—10 mg/kg/day	3 weeks	C57BL/6J mice	↓ Adipocyte size, CSL ↓TNF-α ↓CD68, MCP-1 e p38 ↑AKT
Gambaro et al. [83]	Spexin—29 μg/kg/dia	10 days	Swiss Mice	↓ TNF-α, IL-6, IL-1β, M1a Ly6C-, CD11c, CD11b ↑CD206 ↑M1/M2 reason
Chen et al. [84]	Sea cucumber saponin liposomes—0,45 mg/mL	8 weeks	C57BL/6J mice	↓ Infiltration of macrophages ×℗ ERK ↓TNF-α and IL-6

↑ Increase ↓ Decrease × Inhibit ◊ Stimulate ℗ Phosphorylation

COX2: cyclooxygenase enzyme. TNF-α: tumor necrosis factor alpha. PPAR-γ/2: peroxisome proliferator-activated gamma receptors. CEBP-α: CCAAT-enhancer-binding proteins. SREBPc: sterol regulatory element binding proteins. TLR2/4: toll-like receptors. MyD88: myeloid differentiation primary response gene 88. JNK: c-Jun N-terminal kinases. iNOS: induced nitric oxide synthase. MGL-1: macrophage galactose-type lectin 1. NOX4: NADPH oxidase 4. FoxO1: Forkhead Box O1. STAT3: signal transducer and activator of transcription 3. NLRP-3: NLR family pyrin domain containing 3. FAS: Fas cell surface death receptor. INFα/β: interferons. IL-1, IL- 6, IL-10, IL-13: interleukins. MCP-1: monocyte chemoattractant protein-1. NF-kB: nuclear factor kappa B. CLS: crown-like structures. LPL: lipoprotein lipase. IRS-1: insulin receptor substrate 1. GLUT-4: glucose transporter. LXB4: lipoxin A4. ARG-1: arginase 1. AMPK: AMP-activated protein kinase. UCP-1/2/3: uncoupling protein. SGLT2: sodium/glucose cotransporter 2

### Mechanisms of action of anti-inflammatory agents in the adipose tissue

In the collected data, most anti-inflammatory agents generated outcomes that favored the reduction of pro-inflammatory markers expression by decreasing the infiltration of macrophages in white adipose tissue (WAT) and polarization of M1-type macrophages to M2.

Therefore, because obesity is a disease of multifactorial origin associated with inflammation, the treatment of obesity becomes a challenge. In many cases, lifestyle changes do not present themselves as lasting tools for weight loss. Thus, drug therapy, such as anti-inflammatory agents that complement the caloric deficit and the practice of physical activity, might be used [85].

Considering the class of drugs, Hsieh et al. [45] evaluated the effect of a selective COX-2 inhibitor (celecoxib or mesulid) on inflammation. The study demonstrated that the inhibition of the *COX-2* gene significantly reversed adipocyte hypertrophy, macrophage infiltration, and the genetic expressions of *TNF-α*, *PPAR-γ*, and *CCAAT-enhancer-binding proteins* (C/EBP-α) in epididymal adipose tissue. In contrast, Carvalho et al. [86] evaluated the effect of treatment with a purified tamarind trypsin inhibitor (TTIp) on metabolic changes in obesity. The results showed that the treatment reduced the expression of the TNF-α gene and protein, with no relative effects on the *PPAR-γ* mRNA expression, which indicates that the reduction occurred in a PPAR-γ independent pathway.

Ma et al. [46] used the drug Dextran (D-70) and observed that receptor-mediated M1 macrophages endocytose the Dextran conjugates (D-rad and D-PEG-rad), and free dexamethasone is released into cells. Dexamethasone then binds to the glucocorticoid receptor, inhibiting the transcription of pro-inflammatory genes, decreasing adipocyte hypertrophy, and consequently reducing the production of MCP-1. This protein is responsible for recruiting monocytes and releasing cytokines and chemokines locally through the NF-κB activation. Thus, it reduces the MCP-1 production, decreases inflammation in the adipocyte, decreases TNF-α, IL-6, and MCP-1, size of adipocytes, and crown-like structures (CLS), suggesting a reduction in infiltrated macrophages.

Ndisang & Jadhav [47] observed that the administration of the drug Hemin induced an increase in the release of adiponectin and the activation of AMPK, which stimulates the translocation of GLUT-4, being associated with increased protein expression of IRS-1, PKB/AKT. These factors caused a decrease in oxidative/pro-inflammatory transcription factors NF-κB, AP-1, and JNK, reducing TNF-α IL-6 and IL-1β, MCP-1, and ICAM-1 in the adipose tissue.

Furuya et al. [12] demonstrated that atorvastatin suppressed both IKK-β and IKK-α phosphorylation, decreasing the expression of NF-κB target genes such as *TNF-α* and *IL-6*. Increased GLUT-4 gene and protein expression were also observed.

Okada et al. [48] evaluated the pioglitazone’s action, a thiazolidinedione (TZD) in KKAy mice. The results showed that pioglitazone regulated the production of lipoxin B4 (LXB4), a lipid mediator, in adipocytes, decreased Prostaglandin 2, and induced the mRNA expression of *IL-10*, *IL-13*, and *Arg-1*, concluding that this mediator may have an anti-inflammatory effect on adipose tissue. Xu et al. [49] used Empagliflozin, also of the TZD class, and SGLT2 inhibitor. The drug acted by inhibiting SGLT2 and activating the AMPK pathway, consequently increasing the mRNA expression of *adiponectin* and *UCP-1*. Thus, reducing CLS and pro-inflammatory gene factors such as *leptin* and *F4/80* were also observed.

Prabhu et al. [50] evaluated the action of dextran-based dexamethasone (ND) conjugates in an obesity model. It was observed that ND promoted the marked regulation of inflammation markers and lymphocyte infiltration (*TNFα*, *CD68*, *CD11c*, *CD8a*, *CCR5*, *CD64*, *F4/80*, *CCL3*), as well as reduced expression of metabolic markers (*ACACA*, *UCP3*, *PCK1*, and *IRS2*). A reduction in pro-inflammatory macrophage markers (*IL-6*, *FASL*, *CCR5*, *CCL2*, *NOS2*, *IFNG*, *FCGR1*) and inflammation markers (*TNFα*, *IL-6*, *IL-1B*, *CCR5*, *CCR2*, *CCL2*, *FCGR1*, *NOS2*), while the macrophage marker M2 (*CD206*) increased substantially with ND treatment. Together, these results point to a change in the phenotype of macrophages from M1 to M2 more expressively for ND.

Zhou et al. [51] analyzed a peptide-1 (GLP-1) (Ex-4) 2 -Fc analog in anti-inflammatory functions in adipose tissue. The treatment reduced the expression of *IL-1β*, *TNF-α*, *IL-6*, and *MCP-1*, in addition to the mRNA levels of the cytokines *IL-4* and *IL-13*. M1 macrophage reduction and M2 macrophage increase were also observed. Besides, the expression of their relative markers *CD11b*, *F4/80*, *iNOS*, and an increase in *CD206*. When evaluating the inflammatory signaling pathways in adipose tissue, the data showed that the treatment affected the gene expression of the *AMPK* pathway, reducing the phosphorylation of NF-κB and p38, in addition to the expression and secretion of leptin. These results suggest that (Ex-4)2-Fc suppresses inflammatory signaling pathways, inhibiting NF-κB, p38, and AMPK phosphorylation due to reduced leptin levels in adipose tissue.

Considering natural agents, Lee et al. [52] evaluated the effects of treatment with Metabolaid® (MetA), a combination of lemon verbena and hibiscus flower extracts, on obesity. The treatment decreased the genes related to adipogenesis, *CEBP/α*, *PPARγ*, and *SREBP-1c* in AT. The results also showed an increase in the mRNA expression of the *UCP1* and *UCP2*, which can contribute to the darkening of WAT, promoting thermogenesis, thereby increasing energy expenditure. Besides, the treatment reduced the pro-inflammatory cytokines TNF-α and IL-6. Also, an increase in the phosphorylation of AMPK was observed, indicating that the activation of AMPK may be responsible for reducing adiposity.

Fenni et al. [53] evaluated the supplementation of lycopene and tomato powder in inflammation induced by obesity. Supplementation reduced adipocyte hypertrophy and *PPAR-γ* gene expression, which is considered a key regulator in adipogenesis, thus explaining the reduction in adiposity in supplemented mice. A reduction in the transcription factor SREBP-1c and the *FAS* gene was also observed. The results also showed a decrease in gene pro-inflammatory cytokines (*TNF-α*, *IL-6*) and chemokines (CCL2 and CCL5), which are explained by reducing phosphorylation of p65 and IkB, two important factors of NF-kB signaling. The results suggested that these compounds’ anti-inflammatory effect on adipose tissue results from their ability to inhibit NF-κB signaling in adipose tissue.

Abu Bakar et al. [54] investigated the metabolic effects of Celastrol on the inflammatory response in adipose tissue. When evaluating the mRNA expression of pro-inflammatory genes, it was found that the treatment repressed the expression *of IL-6*, *TNF-α*, and *MCP-1* in adipose tissue. Besides reducing the phosphorylated activity of NF-κB, mRNA expressions of *iNOS* and *CD11c* restored the level of *Arg1*. These results indicate that the improvement in the inflammatory state promoted by Celastrol is associated with a reduction in NF-κB phosphorylation and the polarization of M1-type macrophages in adipose tissue.

Alsaggar et al. [55] in their study aimed to evaluate the anti-inflammatory and antioxidant effects of Silibinin in an obesity model. The treatment suppressed fat accumulation and adipocyte hypertrophy and reversed the pro-inflammatory to anti-inflammatory gene expression profile. The researchers verified the reducing *F4/80 and CD11c expression*, suggesting a reduction in the infiltration of the immune cells in the AT, in inflammatory cytokines *TNF-α* and *IL-6* expression, and regulation of the IL-10 and *adiponectin* expression. These data indicate that silibinin acts by blocking inflammation in adipose tissue, which can be explained by the inhibitory effects of silibinin on NF-κB.

Han et al. [56] aimed to explore the underlying effects of Berberine on obesity-induced inflammation. The results showed that Berberine reduced the mRNA expression of *IL-6* and *MCP-1* and prevented the increased expression of *F4/80* and *TNF-α*. The treatment also increased *IL-4*, *CD206*, and *ARG1*, M2 macrophage markers. Flow cytometry data also indicated blockage of macrophage infiltration into adipose tissue and an increase in the M1 to M2 macrophage ratio. Furthermore, Berberine restored CD163 expression and decreased CD68 expression, indicating suppression of macrophage infiltration and promotion of M2 polarization.

Choi et al. [57] investigated the effect of Indole-3-carbinol, a natural compound found in brassica, on adipose tissue inflammation. Supplementation increased the mRNA expression of Sirtuin 1 (*SIRT1*) and Coactivator 1α (*PGC1α*), leading to the expression of the *UCP1* and *UCP3* genes, which are essential factors in thermogenesis. The results demonstrate that the possible mechanism of attenuation of the inflammation was suppressing the pro-inflammatory cascades mediated by Toll-2 and 4 type receptors (TLRs, toll-like receptors), considering the phosphorylation levels of these pathways were reduced in adipose tissue. When stimulated, these pathways recruit adapter molecules, such as myeloid differentiation primary response gene 88 (*MyD88*). In the study, these levels, together with their target cytokines (*TNFα*, *IFNβ*, and *IL-6*) genes, were reduced, confirming the decrease in inflammation dependent on these pathways.

Ma et al. [58] evaluated the anti-obesity impact of spermidine. The results showed that the treatment reduced the size of adipocytes and the number of CLS. Decreased infiltration of F4/80 macrophages and attenuation of phosphorylation of p38 AMPK and p65 NF-kB were also observed. Furthermore, the treatment reduced the mRNA expression of *SREBF1c*, *FAS*, and *PPARγ* in the same way that it reduced the expression of *IL-1β*, *IL-6*, *iNOS*, *MCP-1*, *CCL5*, and *leptin*. It was observed that the reduction of the inflammatory profile might have been regulated through the inactivation of NF-kB and AMPK, which has been reported as a regulatory kinase of NF-kB.

Wei et al. [59] investigated docosahexaenoic acid (DHA, 22:6) and eicosapentaenoic acid (EPA, 20:5) in the treatment of obesity in mice, using doses ranging from 1 to 4% (w/w). The results showed a reduction in the size and number of adipocytes, attenuating the inflammatory infiltration in the adipose tissue due to the decrease in *IL-6*, *TNF-α*, *CD11c*, and the increase in the expression of *CD206*, a marker of macrophage M2. *FAS* and *ACC reduced* expression, an increase in *adiponectin*, and an inhibition of the increase in *leptin* were also observed. The treatment also showed that EPA 2% might have a regulatory role of *GPR120*, and EPA 1% and DHA 4% may have regulatory roles in inflammation through PPARγ, independent of GPR120, stimulating the dimming of WAT. In contrast, 1% DHA and 4% EPA exerted regulatory effects by mechanisms independent of GPR120 and PPARγ.

Cho et al. [60] evaluated the effects of treatment with Carvacrol. The results showed that the treatment reduced the expression of pro-inflammatory cytokine genes (*TNFα* and *IFNα*), *leptin*, and the mRNA and protein of TLR-2, TLR-4 levels, and their molecules (MyD88, Tirap, TRIF, TRAF6, IRF5, and IRF3). TLR-2 uses the MyD88-dependent pathway, using TRAF-6 and IRF5, leading to NF-κB translocation and cooperation. TLR-4, on the other hand, uses TRIF to activate NF-κB in a mechanism dependent or independent of TRAF-6, leading to phosphorylation of IRF3. The reduced mRNA and protein levels in the treated adipose tissues explain the decrease of pro-inflammatory cytokines. It suggests that meta-inflammation was reduced by inhibiting the TLR-2 and TLR-4 pathways.

Lee et al. [61] observed that treatment with black raspberry oil reduced mRNA and protein expression of TLR-4 and NF-κB and the levels of mRNA of pro-inflammatory cytokines (*TNFα* and *IL-6*) and macrophage markers (*F4/80*, *CD68*, *MCP-1*). Furthermore, there was an increase in mRNA levels of anti-inflammatory markers *IL-10*, *Arg1*, and macrophage galactose-type lectin 1 (*MGL1*) in adipose tissue. Thus, this compound’s possible mechanism of action is by inhibiting TLR-4.

Kim et al. [62] evaluated the effects of resveratrol. The results showed a decrease in mRNA levels of several adipogenic transcription factors, including *PPARγ2*, *C/EBPα*, and *SREBP-1c*, while the reduction of ERK phosphorylation was also observed through protein analysis. The mRNA *TLR-2* and *TLR-4* levels were reduced, as well as the *MyD88*, *TIRAP*, *TRAF-6*, and *TRIF* genes expression as the pro-inflammatory transcription factors *IRF3*, *IRF5*, and *NF-kβ*. These findings, as previously mentioned, justify the reduction of the gene expression of the pro-inflammatory cytokines *TNFα*, *IFNα*, *INFβ*, and *IL-6*, by blocking the pro-inflammatory signaling cascades mediated by TLR-2 and TLR-4.

Oliveira et al. [63] tested the effects of diets containing α-linolenic (ω3) or oleic (ω9) fatty acids. The treatments reduced the expression of the pro-inflammatory markers IL-1β, p-IKK, p-IκBα, TNFα, and p-JNK, and mRNA expression *IL-6*, and the increased expression of *IL-10* mRNA. Activation of the G protein-coupled receptor (GRP120) was also reported. This activation results in the recruitment of β-arrestin and, consequently, in the impairment of inflammatory signaling through TAB1/2, an important transduction pathway for receptors such as TRL-4, thus inhibiting its inflammatory signal. This mechanism explains the reduction in the pro-inflammatory markers’ expression, which is an important way of suppressing inflammation using fatty acids.

Ye et al. [64] evaluated the treatment with Berberine, which inhibited the *MCP-1*, *IL-6*, and *TNF-α* mRNA expressions in adipose tissue, inhibited the phosphorylated activation of JNK, IKKβ, AKT, and reduced the expression of the p65 subunit NF-κB. The M1 macrophage cells (F4/80, CD206) reduction and the M2 (CD11c) increase were demonstrated, demonstrating that the treatment decreased the accumulation of M1 macrophages and the high ratio of M1 and M2 macrophages in adipose tissue. In this way, the treatment inhibits the polarization and activation of M1 macrophages and, consequently, the inflammatory state.

Gao et al. [65], using rutin, observed decreased adipocyte hypertrophy and CLS. As well as reduced mRNA levels of *TNF-α* and *MCP-1*, and reduced gene expression *F4/80*, *CD11c*, and *CD68*, which are markers of pro-inflammatory macrophages and activation markers of pro-inflammatory macrophages and gene activation of CD206 and *Arg-1*. Rutin suppressed the process of infiltration and aggregation of macrophages around necrotic adipocytes, reducing the development of CLS and chronic inflammation, suggesting the anti-obesity effect of this antioxidant. The increase in *Arg-1* is associated with a higher proportion of M2 macrophages, characterized as an anti-inflammatory.

Kang et al. [66] evaluated the treatment with capsaicin, which caused a reduction in the genetic and protein expression of MCP-1, TNFα, IL-6, and leptin, and increased the mRNA expression of *adiponectin*. Besides, the mRNA expression of the potential transient vanilloid type-1 receptor (TRPV-1), a specific receptor for capsaicin, was evaluated. An increase in *TRPV-1* mRNA expression in adipose tissue has been demonstrated, indicating that TRPV-1 is involved in the inhibitory effect of dietary capsaicin on obesity-induced adipose tissue inflammation.

Veerami et al. [67] used *Lavatera critica* (LC), a green herb used in folk medicine. The treatment reduced pro-inflammatory genes *TNF-α*, *IL-1β*, *IL-6*, *iNOS*, *MCP-1*, and *CD11c*. In contrast, the expression of the anti-inflammatory genes *Ym-1*, *ARG1*, and *Clec7a* was increased. The attenuation of enzymatic (SOD and GPx) and non-enzymatic (total proportion of GSH and GSH/GSSG) attenuation was also observed. Thus, the compound acts by reducing inflammation and damage from oxidative stress.

Yang et al. [68] evaluated the effects of bitter melon (*Momordica charantia*), which reduced the mRNA expression of *TNF-α*, *IL-6*, and *CCL2* in adipose tissue and reduced phosphorylation of NF-kβ (p65) and JNK. These pathways are possibly the main ones responsible for suppressing the inflammation used by this compound.

Ying et al. [69] used Interferon tau to treat inflammation in obesity. The treatment suppressed the activation of NF-κB, thus evidenced by the lower p65 phosphorylation and activity of the JNK pathway, evidenced by the decrease in p-JNK. The *IL-1β*, *IL-6*, and *TNF-α* genes’ expression were also reduced, and there was an increase in the anti-inflammatory cytokine *IL-10* gene expression. It was also observed that the number of pro-inflammatory M1 macrophages was drastically reduced, in contrast to the significant increase in M2 macrophages, suggesting regulation in the polarization of macrophages.

Similarly, Zhou et al. [70] using Catapol, a bioactive compound found at the root of glutinous Rehmannia, observed pro-inflammatory M1 gene expression, such as *TNF-α*, *IL-6*, *IL-1β*, *MCP-1*, *iNOS*, and *CD11c* had been reduced. The expression levels of M2 anti-inflammatory genes, such as *ARG1*, *Ym-1*, *IL-10*, *MGL1*, and *Clec7a*, were significantly increased. There was also a reduction in CLS and F4/80, proving the decrease in macrophage infiltration. These results indicate that the catapol can redirect M1 macrophages to an M2 polarized state, explaining the inflammatory state’s reduction. A significant decrease in the JNK and IKKβ phosphorylation was also mentioned, minimizing the activation of NF-κB p50, indicating that treatment through these pathways contributes to the AT’s protective effect against inflammation.

Subramanian et al. [71] evaluated the anti-obesity effects of *Terminalia chebula* fruit extract. It was observed that the treatment regulated the protein levels and gene expression of FAS, PPARα, and CPT-1α, reducing *TNF-α* and *IL-6* cytokines, decreasing *adiponectin* levels, and increasing *leptin* levels. These results indicate the protective role of the extract in the development of inflammatory responses. These effects are related to the suppression of lipogenesis by reducing *FAS* and the increase in the oxidation of fatty acids by PPARα and CPT-1α activity, culminating in the anti-inflammatory responses.

Yu et al. [72] investigated the effects of sarsasapogenin (ZGY) on adipose tissue inflammation. The results showed that the treatment suppressed the expression of the pro-inflammatory genes *TNF-α*, *IL-1β*, *IL-6*, *MCP-1*, *Nos2*, *COX2*, and *adiponectin*, and a simultaneous increase in *Arg1*, *Ym1*, *Fizz1*, and *IL-10*. Inactivation of inflammatory signaling pathways IKK, NF-κB, and JNK was also observed and increased IκB-α protein level in adipose tissue. The treatment reduced the percentage of F4/80 cells, the expression of *F4/80* and *CD68*, M1 markers, and increased the levels of genes associated with M2 *IL-10*, *Ym1*, and *Fizz1*. These results indicate that the mechanism of action attenuating inflammation occurs through the inactivation of the IKK, NF-κB, and JNK signaling pathways.

Bashir et al. [73] evaluated the effects of fish oil on inflammation. A decrease in NF-κB/p65 levels was observed, as well as pro-inflammatory cytokines (TNF-α, IFN-γ, IL-1β, and IL-2) expression and an anti-inflammatory increase (IL-4 and IL-10). The cytometric analysis demonstrated a decrease in TLR-4, which may be responsible for decreasing the inflammatory state. Besides, an increase in *ARG1* mRNA was observed, suggesting a greater polarization of M2 macrophages.

Bettaieb et al. [74] using flavan-3-ol (-)—epicatechin (CE) showed that the treatment decreased the F4/80 protein and reduced the phosphorylation of IKK, IκB, and p65, inhibiting the NF-κB signaling pathway. Besides, a reduction in TNF-α and MCP-1 was seen, and a decrease in protein expression of NADPH Oxidase 4 (NOX4) and p47 means oxidative stress attenuation. Thus, inhibition of the NOX4 pathway led to attenuation of JNK and IKK/NF-κB, justifying the reduction of pro-inflammatory cytokines and the recruitment of M1 macrophages.

Guo et al. [75] evaluating the effects of anthocyanin cyanidin 3-glucoside, noticed a reduction in the macrophage markers F4/80 (Emr1) and Cd11c and a lower infiltration of macrophages in TA. There was also a decrease in mRNA expressions of *TNF-α*, *IL-6*, and *MCP-1* and the activation of JNK and Forkhead Box O1 (FoxO1). Thus, it was suggested that anthocyanins blocked the activation of JNK/FoxO1, suppressing the expression of pro-inflammatory cytokines and improving the inflammatory condition.

Kwon et al. [76] evaluated the mechanisms and effects of *Coptidis rhizoma* on obesity-induced inflammation. The results showed that the treatment reduced the percentage of M1 CD11c macrophages and increased the M2 CD206 macrophages. A reduction in the expression of *TNF-α*, *F4/80*, *CCL2*, *CCL4*, and *CCL5* was also observed. These results indicate that Coptidis Rhizoma reduces chronic low-grade inflammation by reducing macrophage infiltration and shifting from M1 to M2 macrophage phenotype.

Lee et al. [77] evaluated the effects of *Broussonetia papyrifera* root bark extract in treating inflammation associated with adipose tissue. After treatment, the results showed a decrease in NF-κB phosphorylation and an increase in AMPK phosphorylation in WAT, and a reduction in the expression of pro-inflammatory genes *IL-1β* and *iNOS*. These findings indicated that the treatment inhibited the inflammatory responses induced by TNF-α by suppressing the activation of NF-κB, proposing that this extract acted by inhibiting this pathway by blocking the degradation of IκB.

Perdicaro et al. [78] used quercetin in the treatment of adipocyte hypertrophy and inflammation. The results indicated that supplementation reduced the size of adipocytes and modified the levels of proteins involved in angiogenesis and adipogenesis, reducing HIF-1α and increasing VEGF-A and VEGF-R2. An increase in the regulation of protein levels of PPARγ, C/EBPα, adiponectin, and FAS was also observed, indicating the activation of adipogenesis. Supplementation also reduced the protein expression of TLR4, CD68, MCP-1, and p-JNK, indicating a reduction in macrophage infiltration in adipose tissue. These results suggest that quercetin can stimulate a healthy expansion in WAT, promoting angiogenesis and adipogenesis.

Ramalho et al. [79] evaluated eicosapentaenoic acid (EPA) in regulating metabolic and inflammatory pathways through the modulation of transcripts and miRNA in TAB. The results showed a lower expression of adipocyte hypertrophy and inflammation, such as *Arachidonate 5-lipoxygenase (Alox5)*, *Myd88*, *Stat1*, *Ccr5*, and *Card11* lower production of LTB4, consistent with the reduction of *Alox5*. These data suggest that EPA can downregulate inflammatory pathways. However, it negatively affects the LTB4 synthesis machinery, as EPA and other free fatty acids (FFA) are regulated by EPA supplementation in hypertrophic adipose tissue. When analyzing the effects on the microRNAs involved in inflammation, it was observed that EPA reduced the expression of miR-146b and miR-125b-5p, indicating that this reduction would probably be through the decrease of TLB4. It was also found that the regulation of miR-30a-5p and miR-143-3p indirectly decreases inflammatory targets, such as c-jun and c-fos (part of the MAPK signaling pathway).

Regarding synthetic materials, Dinh et al. [80] evaluated Methyl Bardoxolone in the treatment of obesity. Decreased F4/80, protein expression of TNF- α, and protein levels of molecules activated by oxidative stress (STAT3, Akt, ERK, and JNK) have been reported. Thus, the authors discussed that this compound has an antioxidant effect, responsible for reducing obesity and the pro-inflammatory state.

Wang et al. [81] used sodium butyrate to treat inflammation in adipose tissue, and this compound reduced the *CD68*, *INF-γ*, and *MCP-1* mRNA, the number of F4/80 cells, and the *IL-1*, *IL-6* gene expression, and *TNF-α*. The treatment also inhibited the expression of *NLRP3* and caspase-1 mRNA. Thus, one of the possible mechanisms by which the suppression of inflammation occurs is the inhibition of this pathway.

Bak et al. [82] evaluated the Licochalcone F1’s action, observed a reduction in the adipocytes’ size and the infiltration of macrophages. The results also showed decreased *CD68*, *TNFα*, and *MCP-1* expression genes, increased Akt phosphorylation and reduced p38 phosphorylation. Therefore, the modification in the adipocytes’ structure may have been responsible for reducing pro-inflammatory cytokines. Besides, MAPK proteins, such as p38, have an essential role in regulating these cytokines. The phosphorylation of p38 MAPK in adipose tissue indicates an inhibitory effect on p38 MAPK, possibly leading to anti-inflammatory responses.

Gambaro et al. [83] evaluated the role of spexin as a modulator of the immune response of adipose tissue in obesity. The treatment reduced adipocyte size, restored leptin expression levels, reduced *TNF-α*, *IL6*, and *IL-1β* levels, and increased *CD206* levels. Besides, CD11b, Ly6C, and M1 CD11c monocytes were reduced. When comparing macrophage populations, it was found that M2 Ly6C macrophages were more abundant after treatment, demonstrating the modulation of inflammation in WAT, improving the M1/M2 ratio.

Chen et al. [84] used a treatment with marine cucumber saponin liposomes. The treatment reduced the mRNA expressions of pro-inflammatory cytokines *TNF-α* and *IL-6* and decreased macrophage infiltration in the adipose tissue, which decreased the *F4/80* and *CD11b* mRNA expression. Increases in prostaglandin and ERK phosphorylation inhibition have also been observed, probably one of the leading causes of reduced pro-inflammatory markers.

Additionally, it was observed that the studies included in this review also emphasized, as possible explanations for the improvement in the inflammatory profile, the action of anti-inflammatory agents on adipocyte hypertrophy/hyperplasia, as well as on the beige of adipose tissue. Therefore, studies have highlighted these factors as responsible for thermogenesis and weight and body fat changes.

In some studies, the authors reported that the browning of white adipose tissue occurred, which increased thermogenesis, energy expenditure, and weight reduction. Besides, reducing M1-type macrophages and increasing M2-type macrophages in fat and liver impact reduced insulin resistance and reduced inflammation, especially in adipose and liver tissue [50, 51, 52, 56–59, 64–66, 69, 70, 72, 75, 76, 83].

Based on this, considering the various regulatory functions of adipose tissue, and according to the studies mentioned above, the expansion of this tissue stands out for its impact on adipose cell inflammation, as well as on body energy balance and glucose homeostasis. Such dysfunctions are associated with the risk of several chronic diseases, causing systemic effects that include increased body weight, insulin sensitivity, and metabolic disorders that impact the body in general. Thus, this review presented the anti-inflammatory agents that act directly on adipose tissue in preclinical analyses, can serve as a basis to guide clinical studies, and, in the future, these agents will be used in more comprehensive treatments that aim at targets related to regulatory functions of the AT.

However, suppression of adipocyte inflammation can result in adipose tissue dysfunction and promote insulin resistance. Zhu et al. [87] evaluated the relative contributions related to inflammation of adipocytes and macrophages to insulin sensitivity. The results showed that, even with the reduction of adipocytes, body weight, and suppression of inflammatory pathways (TNFα and IL-1β), glucose tolerance was intensified, increasing insulin resistance, fatty liver, and reducing adiponectin. This suggests that despite the beneficial effects on weight gain, suppression of adipocyte inflammation impaired adipose tissue function and promoted insulin resistance.

Asterholm et al. [88] evidenced that pro-inflammatory signaling in adipocytes was essential for good remodeling and expansion in adipose tissue. The researchers used mice with a specific reduction in the pro-inflammatory potential in adipose tissue, which, when exposed to a high-fat diet, had tissue expansion prominently affected. These effects were related to decreased intestinal barrier, increased hepatic steatosis, and metabolic dysfunction and led to increased accumulation of ectopic lipids, glucose intolerance, and systemic inflammation. In conclusion, inflammatory signaling in adipocytes is an adaptive response that allows the safe storage of nutrients and contributes to a visceral deposition barrier that filters gut-derived endotoxins.

Thus, even though the present review shows and gathering anti-inflammatory agents that acted directly on adipose tissue in preclinical studies with numerous positive effects, it is still necessary to evaluate in a broad and detailed way. However, this information can serve as a basis to guide clinical studies. In the future, these agents will be used in more comprehensive treatments that target related to regulatory functions of the AT, without causing harm, for example, insulin intolerance or resistance. It also highlights the importance of future studies in women (feminine gender), whether preclinical or clinical, to investigate how these anti-inflammatory agents act on the adipose tissue of this gender.

**Fig 3:**
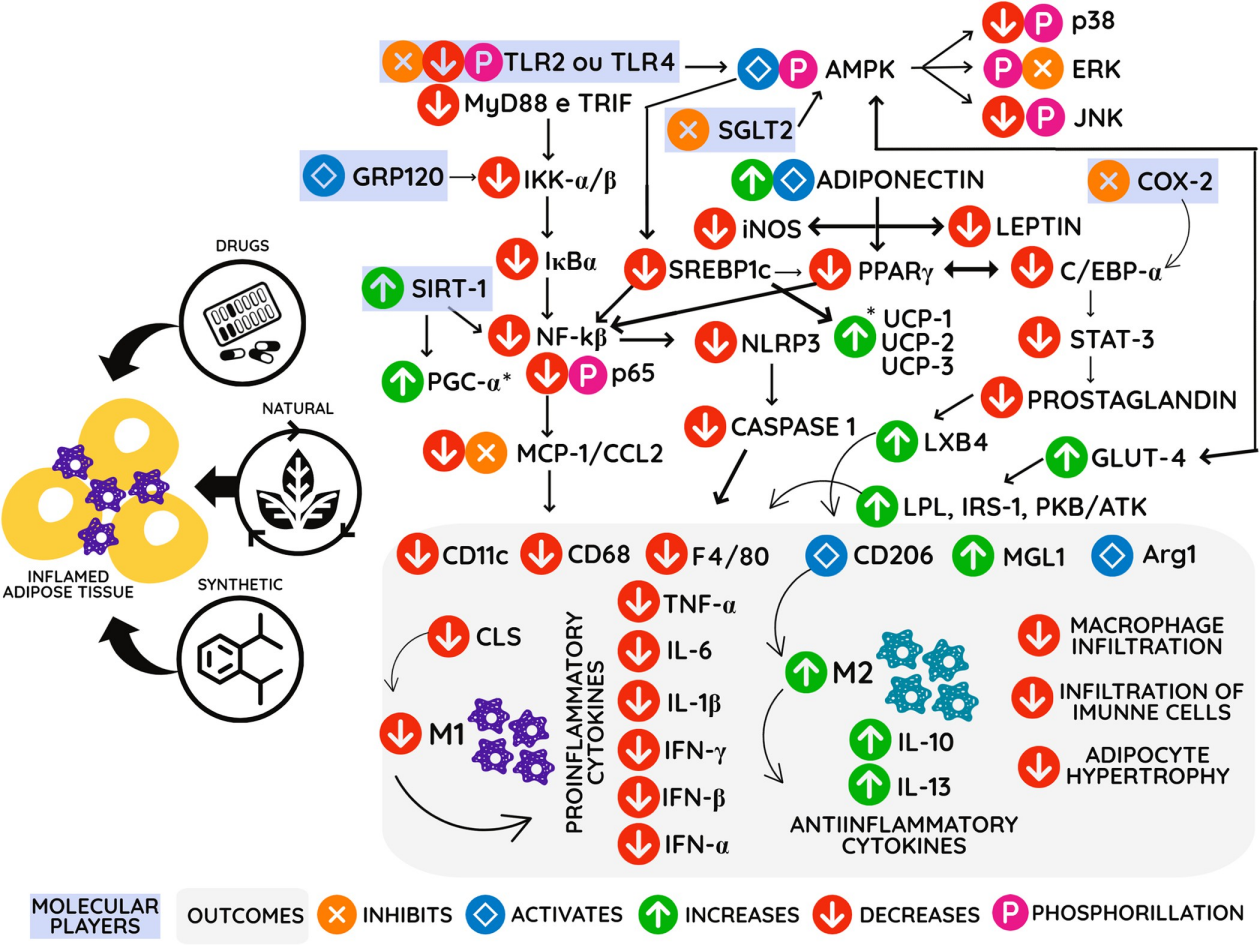
Mechanisms of action of anti-inflammatory agents in adipose tissue on the expression of genes and proteins in a hypothetical cell. Anti-inflammatory agents (Drugs, natural and synthetic compounds) act in several ways that cross and culminate in reducing infiltration of immune cells, reducing the pro-inflammatory profile, and changing the M2 and M1 polarization, inducing an anti-inflammatory profile and suppressing inflammation in obesity. COX2: cyclooxygenase enzyme. TNF-α: tumor necrosis factor-alpha. PPAR-γ/2: peroxisome proliferator-activated gamma receptors. CEBP-α: CCAAT-enhancer-binding proteins. SREBPc: sterol regulatory element-binding proteins. TLR2/4: toll-like receptors. MyD88: myeloid differentiation primary response gene 88. JNK: c-Jun N-terminal kinases. iNOS: induced nitric oxide synthase. MGL-1: macrophage galactose-type lectin 1. NOX4: NADPH oxidase 4. FoxO1: Forkhead Box O1. STAT3: Signal transducer and activator of transcription 3. NLRP-3: NLR family pyrin domain containing 3. FAS: Fas cell surface death receptor. INFα/β: interferons. IL-1, IL- 6, IL-10, IL-13: interleukins. MCP-1: monocyte chemoattractant protein-1. NF-kβ: nuclear factor-kappa β. CLS: crown-like structures. LPL: lipo-protein lipase. IRS-1: insulin receptor substrate 1. GLUT-4: glucose transporter. LXB4: lipoxin A4. ARG-1: arginase 1. AMPK: AMP-activated protein kinase. UCP-1/2/3: uncoupling protein. SGLT2: sodium/glucose cotransporter 2.

Finally, the studies included in this systematic review made it possible to infer that a large part of the bioactive compounds, drugs, and synthetic compounds act in metabolic pathways that culminate, mainly in reducing the expression of inflammatory cytokines. This action favors a reduction in macrophages’ infiltration in white adipose tissue and causes a polarization process of macrophages of type M1 to M2 (Fig 3).

The mechanisms highlighted in this review were proposed only considering studies conducted with male animals. It is known that male mice have greater weight gain than females and, therefore, are ideal for comparison focusing on white adipose tissue. In addition, several hormonal factors can regulate inflammatory pathways in females and promote differences in metabolic reproduction [89–93]. Therefore, studies involving the female animals were excluded, thus following the exclusive determination activity, since the study’s primary objective covers the specific antiadipogenic mechanisms.

The limitations of this review are related to not performing a meta-analysis, considering the clinical, methodological, and statistical heterogeneity of the results and the variability of the articles included. It is also important to highlight the methodological quality of the articles, as many studies do not present items at risk of bias, leaving the evaluation less clarified. However, the results portray scientific evidence of high relevance concerning the topic addressed.

## Conclusions

Several anti-inflammatory agents have been evaluated for mechanisms of action to suppress inflammation in adipose tissue. We can observe that drugs, natural and synthetic compounds act in several ways that meet and unite to suppress inflammation. Among the primary outcomes found a reduction in anti-inflammatory cytokines, adipocyte hypertrophy, which ultimately cause a decrease in macrophage infiltration, and a change in the polarization of M1→M2 macrophages, inducing an anti-inflammatory state and suppression of inflammation in the AT.

Therefore, using these anti-inflammatory agents is relevant to developing new studies and clinical trials aiming at promising new treatments for obesity and metabolic changes resulting from this state of inflammation. Furthermore, it is noteworthy that in two studies presented in this review, suppression of adipocyte inflammation resulted in adipose tissue dysfunction and promoted insulin resistance.

This systematic review synthesizes and schematizes presenting various molecules targeted by anti-inflammatory agents through mechanisms that increase or decrease their expressions at the molecular, systemic, or tissue level. This evidence may improve and choose the anti-inflammatory agent for possible applications aiming at future treatments with specific and promising targets.

## Supporting information

S1 Checklist PRISMA 2020 checklist. (DOCX)
